# Preliminary Validation of Digital Photography to Assess the Home Food Environment

**DOI:** 10.3390/ejihpe13070093

**Published:** 2023-07-12

**Authors:** Daniela Quan, Isaac Michael, Elizabeth Gollub

**Affiliations:** 1 School of Nutrition and Food Sciences, Louisiana State University, Baton Rouge, LA 70803, USA; dquan3@lsu.edu; 2 Department of Experimental Statistics, Louisiana State University, Baton Rouge, LA 70803, USA; imichael@lsu.edu; 3 School of Nutrition and Food Sciences, Louisiana State University Agricultural Center, Baton Rouge, LA 70803, USA

**Keywords:** home food environment, home food inventory, digital photo, validation

## Abstract

The home food environment (HFE), the availability and accessibility of foods and food products within the home, has a strong influence on healthy eating behaviors. Studies assessing the HFE commonly utilize a home food inventory (HFI) for data collection. However, this approach tends to be burdensome for participants. This study validated a low-burden digital photo method for assessing the home food environment and confirmed that this method is preferred by participants. Study participants completed an HFI, submitted photos of household foods, then identified preference for the HFI or photo reporting method. Researchers completed an HFI based on each participant’s photo submissions. Researcher-to-participant and researcher-to-researcher comparisons were made through ANOVA and randomized block analyses to determine concurrent validity and inter-rater reliability. Method preference was assessed using the Z-test. The participant group (N = 53) was predominantly female (68%), young adult (90.5%), and Hispanic or Latino (71.7%). Concurrent validity was initially moderate (ĸ = 0.54); adjustments yielded substantial agreement (ĸ = 0.61). The inter-rater reliability (*p* = 0.98) demonstrated significant consistency among reviewers. The photo-documentation method was found to be valid and preferred (*p* = 0.01) for reporting on the HFE. The photo method can be used advantageously to collect quality data.

## 1. Introduction

The home food environment (HFE) refers to the availability and accessibility of foods and food products within the home. It is an important factor for addressing and supporting healthy eating behaviors [1,2]. Most adults in the US consume approximately 68% of their calories from home food sources, with diets that are, on average, high in fats, sodium, and sweets and low in fruits, vegetables, and whole grains [3]. These unhealthy diets have a major influence on obesity (currently affecting ~42% of US adults [4]) and other chronic health conditions such as diabetes, hypertension, heart disease, stroke, and some types of cancers [4,5]. Since 2020, as a result of the COVID-19 pandemic, adults in the US have been cooking and eating at home more frequently [6]. There has also been an increase in the number of adults attempting to improve their diet [7]. As these shifts occur, the HFE becomes even more significant.

Food intake is a reflection of food availability, which largely comprises household food availability [1,8]. However, food placement within the home also influences food consumption. Among adults, home environments in which high-fat food options have been kept out of sight, while fruits and vegetables were placed within easy reach, have been associated with decreased consumption of fat and increased consumption of fruits and vegetables [9]. This finding suggests the potential to affect a healthier diet by altering the placement of food items or presentation of food choices within the home [10]. The application of *nudging* [11] to encourage healthier food choices within the home can be an effective focus for nutrition education. Thus, there is a need to develop a method of assessing this aspect of the HFE.

Home food inventories (HFIs) are commonly used to document the foods and food products in the home [8]. HFI data can be collected from direct observation by a researcher or from self-reports, which can be burdensome for the reporter and problematic for the researcher [12]. Digital photography, a documentation and reporting method considered user-friendly to both reporter and researcher [8], could provide a functional alternative method of data collection.

A digital photo method to assess food intake was initially developed in 1983 [8] and has been used successfully [13,14]. Over a decade ago, a feasibility study of cellphone camera documentation concluded that this method of reporting was acceptable, simple, and sufficiently valid [13]. Recently, a nutrition education program utilizing principles of choice architecture for strategic placement of food in the home kitchen area applied a digital photo method to documentation of certain aspects of the HFE [15]. To assess changes in the HFE as a program outcome, participants submitted pre- and post-program photos of the contents of their refrigerator and pantries. However, the use of digital photography for assessment of food items or for change in food placement within the home had not been validated.

The purpose of this research was to address a fundamental portion of this gap by validating the photo-method for assessment of the HFE/food items to determine whether it can be a systematic, reliable, objective process. A second aim of this study was to confirm the assumption that study participants would prefer the photo-method to completion of a home food inventory when reporting on the HFE.

## 2. Materials and Methods

Using a home food inventory method (a checklist) as the standard, this study was designed to determine whether the photo method can reliably and accurately capture and communicate information about an individual’s home food environment. This study was approved by the Louisiana State University (LSU) AgCenter Institutional Review Board and conducted from March to August 2022.

### 2.1. Participants and Recruitment

The group of study participants was a convenience sample of adults (eighteen years of age or older) who did their own grocery shopping, had a living space with a standard kitchen (refrigerator, freezer, counter space/preparation area, pantry/food storage cabinets), had access to a camera (phone or otherwise), and had internet access. A recruitment flyer was created to describe the study, list participant inclusion criteria and responsibilities, provide researcher contact information, and display a QR code to initiate the participation process. The flyer was electronically distributed to 3 classes and broadcast to graduate and undergraduate students in the College of Agriculture. It was also announced (electronically) through university student associations, and within 7 communities from around Louisiana or Florida, through nutrition extension programs of the LSU AgCenter and the University of Florida IFAS. Consent was obtained from each participant prior to data collection.

### 2.2. Study Tools and Procedures

Study participants were asked to complete a study survey consisting of a home food inventory (HFI) and several demographic items. They were also asked to submit photos of the contents of their refrigerator, freezer, and pantry using instructions developed specifically for this study. Participants were then asked to report their preference for the inventory or photo method. The entire process was conducted online, and a small incentive was provided to encourage participation.

The study HFI was developed as a checklist of key food/food product/beverage items considered typical for households in the US southern region and relevant to outcome measures for nutrition education programs, interventions, diet quality assessments, or healthy eating campaigns. Checklist items were adapted from the Home Food Availability Inventory Checklist (HFAI-C), although DASH diet indexes and the Healthy Eating Index-2015 (HEI-2015) were compared to further inform item selection. The tools and other items used to develop the study survey were previously validated and/or used with a similar target population [16,17,18].

A total of 46 food items were included and were grouped by food category: total fruits, total vegetables, protein foods, dairy, grains, legumes, miscellaneous, nuts, fats and oils, snacks and desserts, and beverages (Table 1). In each of these 11 categories, several sub-categories of food items were presented. For example, the sub-categories of total fruit were fresh fruits; canned fruits; dried fruits; frozen fruits; and cut, washed, and ready-to-eat fruits. For each checklist item, participants were asked to indicate “yes” or “no” if these items are in the home today/now. If “yes”, the participant was asked to indicate the storage location (refrigerator, freezer, pantry, other) of that food.

Demographic items were included in the participant survey to capture basic participant characteristics in terms of gender, age, race/ethnicity, level of education, level of food security, and neighborhood (e.g., rural, or urban). As drafted, the two-part survey was then subjected to expert review for content, clarity, comprehensiveness, flow/presentation, length, and other suggestions. Based on reviewer comments, revisions were made.

A guidance document was created to help standardize submissions for the study’s photo component. Specific instructions on how to take photos of the refrigerator, freezer, and pantry contents to capture all food items were provided. For example, if refrigerator or freezer drawers were not transparent, an additional photo with an open drawer was requested. Picture angle, orientation, lighting, and photo clarity were emphasized and illustrated. Participants were to submit photos via email; the address was included. The photo instruction document was reviewed externally. To narrow the focus for this preliminary validation study, participants were instructed to exclude foods that were not stored in the kitchen.

The entire data collection process was field tested by 5 volunteers, student or non-student community dwelling adults. Field testers used the flyer QR code to initiate the process. After providing their informed consent, they were e-mailed a link to the participant survey (HFI + demographics). Once this survey was completed and submitted, the field tester was sent the photo instructions. Field testers and later the actual participants were instructed to complete the survey and photo components on the same day and as close to the same time as possible. Once the photos were received, the field tester was sent the final participant study question asking which method (checklist or photo) is preferred for reporting on the food in their home.

The field test volunteers were contacted for their comments on the study process and for their responses to specific questions about clarity, flow/presentation, content perception, and length of the survey and the photo instruction document. Field test volunteers were also asked to provide any additional observations or suggestions. Minor revisions were made to address comments or suggestions from the field testers. None of the field testers were included in the study sample.

### 2.3. Data Analysis

The analysis was geared toward addressing two questions: “can photos of the home food environment provide researchers with the same quality of information as a traditional home food inventory?” and “do participants prefer the photo method for reporting on the HFE?” The first question was accomplished by determining whether different researchers reviewing the same photos would obtain the same information (inter-rater reliability); and whether this information was the same as the information reported by the participant (concurrent validity).

**Figure 1:**
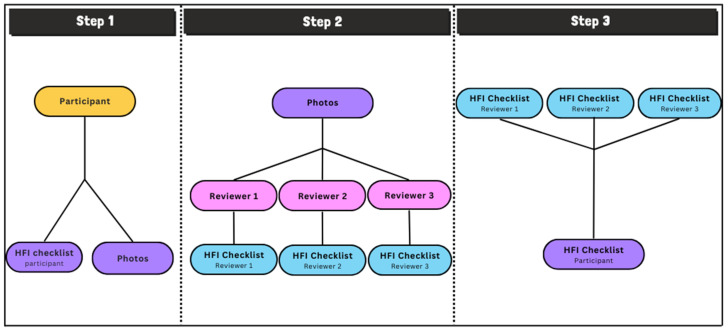
Flow diagram of validation study.

These two components of validity, inter-rater reliability and concurrent validity, were measured as the third step of the three-step validation study process (Figure 1). First, the participant completed the HFI checklist and submitted photos of their refrigerator, freezer, and pantry contents. Second, using the photos provided by the participant, each of the 3 reviewers completed an HFI checklist for each participant. Third, agreement rates between the HFI completed by the participant and the HFI completed by the reviewer and agreement among the reviewers were calculated.

Prior to the first participant review, the 3 reviewers were instructed on the procedure and completed a practice HFI checklist using photo data from one of the test volunteers. The reviewers then completed their reviews independently.

An analysis of variance (ANOVA) and randomized block design were used to simultaneously determine the level of agreement, based on Cohen’s kappa (ĸ), between reviewer and participant (concurrent validity) and among the three reviewers (inter-rater reliability). This was calculated using the formula: ĸ = (observation agreement − experimental agreement)/(1 − experimental agreement), which compared agreement rate for Reviewer 1 on Item 1,…, Item 46; agreement rate for Reviewer 2 on Item 1,…, Item 46; agreement rate for Reviewer 3 on Item 1,…, Item 46, for all participants.

The ĸ statistic was interpreted as follows: ĸ < 0.00 = no agreement, ĸ = 0.00–0.20 = slight agreement, ĸ = 0.21–0.40 = fair agreement, ĸ = 0.41–0.60 = moderate agreement, ĸ = 0.61–0.8 = substantial agreement, ĸ = 0.81–0.99 = near perfect agreement, ĸ = 1.0 = perfect agreement [19].

For the randomized block design, each rater was considered a block from which each of the 46 items from each of the participants were compared. Reviewer–participant agreement (mean; variation) for each HFI item was placed into 1 of 4 categories: (A) low kappa and high range, (B) low kappa and low range, (C) high kappa and high range, (D) high kappa and low range. This analysis permitted the characterization of the reviewer–participant agreement for each of the 46 HFI items and provided an overview of which food items might have diminished the overall agreement.

Items with weak reviewer–participant agreement were further scrutinized. Reviewer–participant data were compiled as numbers and rates of: (1) Items seen in photos by the reviewer and reported by the participant, (2) Items seen in the photos by the reviewer but not reported by the participant, and (3) Items reported by the participant but not seen by the reviewer.

The second question, the method preference among participants, was answered using the Z-test. All computations were performed using SAS statistical software (version 9.4).

## 3. Results

A total of 53 participants completed the study; no one opted out. The participant group was predominantly female (68%), young adult (90.5%), Hispanic or Latino (71.7%). Additional participant group characteristics are presented in Table 2.

Inter-rater reliability and concurrent validity were measured as an integrated analysis. The reviewer–participant agreement, calculated as a ĸ value for each reviewer (R1 = 0.546; R2 = 0.541; R3 = 0.547), represents the agreement mean for each reviewer and all 53 participants. These kappa values were used to determine the Kappa mean = 0.54, moderate agreement among the three reviewer–participants HFI data sets. The *p*-value, *p* > 0.98, demonstrates that there is no statistically significant difference in agreement between reviewers.

**Figure 2:**
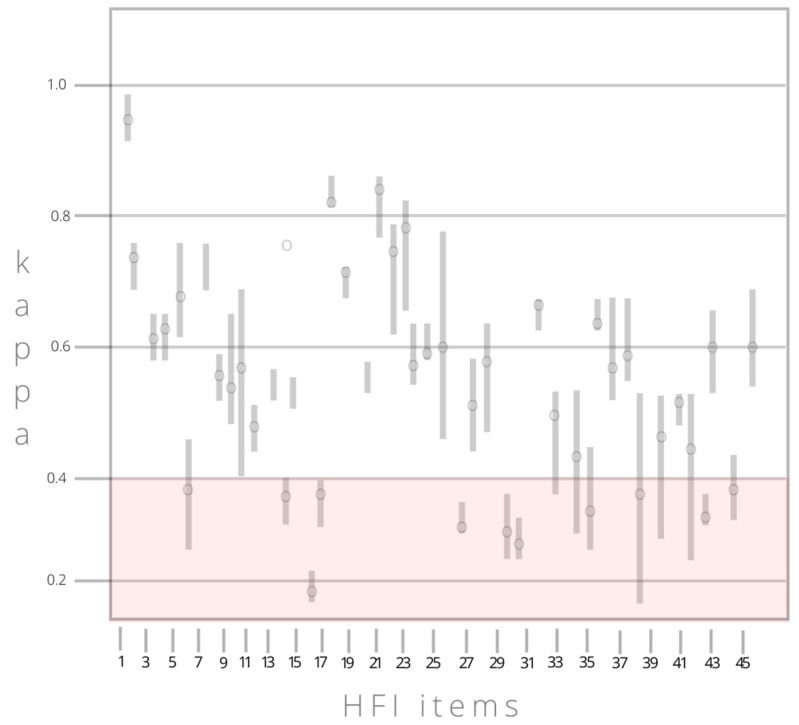
Reviewer–participant agreement, mean and variation, for each of the 46 HFI items.

Each HFI item was also viewed individually. Figure 2 illustrates the reviewer–participant agreement as mean variation and spread within each of the 46 food items. Referring to Figure 2, the food items below ĸ = 0.4 (shaded area) are those that diminished the overall agreement. For these 11 items, there is high variation across reviewers, as with Items 6 (canned vegetables) and 39 (sweet snacks), or low reviewer–participant agreement as with Items 13 (other vegetables), 16 (fresh or frozen fish or seafood), 17 (processed fish or seafood), 27 (whole grains), 30 (fresh or dried legumes), 31 (processed legumes), 35 (nut butters), 43 (non-carbonated sweetened drinks or powders), and 45 (water/carbonated waters).

Each of these 11 items was analyzed individually to assess the basis of the low agreement. Table 3 shows the type and number of occurrences and of reviewer–participant discrepancies compiled for three situations. This analysis suggests that Items 27, 35, 39, and 43 (shaded rows in Table 3) might have been underreported by the participant, while Items 6, 13, 16, 17, 30, 31, and 45 might have been overreported by the participant or not visible (to reviewers) in photos.

An ANOVA was rerun excluding these 11 food items. This increased the kappa values for each reviewer (R1 = 0.613; R2 = 0.615; R3 = 0.605) and the Kappa mean to 0.61 (*p* = 0.94), which is considered substantial agreement.

Among the 53 study participants, 42 (79.2%) preferred the digital photo method to the completion of the home food inventory for reporting on the home food environment. The Z-test was used to determine that the photo method was significantly preferred (*p* = 0.01).

## 4. Discussion

The purpose of this study was to determine whether the photo method can be used as a proxy for a home food inventory, and whether this method can accurately capture and communicate information about an individual’s home food environment. This was achieved by determining overall agreement between the HFI completed by the participant (the standard approach) and the HFI completed by the reviewers using digital photos submitted by participants. As a preliminary validation, the study was limited to kitchen area food storage and to a convenience study population which consisted almost entirely of young adults, most of whom were Hispanic or Latino and female. Within these parameters, results from this study demonstrate that the digital photo method can be a viable and reliable method for reporting on food items within the home.

To date, most assessments of the home food environment, for food availability or quality, have used some version of the self-report checklist [16,20]. The value of the photo method is that it might also be used to assess placement of foods in the home, an important evaluative application when promoting home food environments in which healthier choices are to be easier to locate and reach. The HFI created for this study was based on the standard Home Food Availability Inventory Checklist (HFAI-C) tool [16]. Food items included in the study checklist were selected intentionally to reflect foods common to the region, typically stored in the kitchen area, and relevant to nutrition education program outcomes.

Although the study population was relatively homogeneous, the distribution assumptions associated with the statistical tests used in this study permitted a robust analysis for the 53-participant sample size [21]. The analytic method simultaneously permitted the identification of food items that diminished the overall agreement between the participant and the reviewers. The standard HFAI-C, validated through analysis of Cohen’s kappa coefficient, had an overall *fair to moderate* agreement score when evaluated by food item [16]. In this study, using all 46 HFI items, the ANOVA results indicated *moderate agreement*, which provided numerical support for the validity of the digital photo method. However, certain food items were clear outliers in the data, indicating that modified identification methods might need to be created for higher matching rates in the future. After removal of these outliers, the ANOVA results indicated *substantial agreement*, which strongly indicates that, at least for certain easily identifiable food groups or items, the digital photo method has strong concurrent validity.

Among the 11 food items that were removed, 4 were underreported by study participants (Table 2 and Table 3). Whole grains (item 27), nut butter (item 35), sweet snacks (item 39), and non-carbonated sweetened drinks (item 43) were seen in the photos by the reviewers more frequently than they were reported by the participant. The underreporting of whole grains might reflect participant difficulty determining which products contain whole grains. A recent study evaluating product label comprehension found that 33.8% of participants experienced confusion identifying whole grain products [22]. For the case of nut butter, it is possible that participants reported this food item in the “nuts” category rather than in “nut butter”. In this validation study, sweet snacks and non-carbonated sweetened drinks were more frequently underestimated by the participants. This was noted by other researchers using a home food inventory [23], possibly attributable to social desirability bias, which affects how people self-report their nutritional intake [24,25].

Participants were thought to have overreported seven HFI items. Canned vegetables (item 6), other vegetables (item 13), fresh or frozen fish or seafood (item 16), processed fish or seafood (item 17), fresh or dried legumes (item 30), processed legumes (item 31), and water/carbonated waters (item 45) were reported by the participant more frequently than they were seen by the reviewer (Table 2 and Table 3). It is possible that scenarios other than overreporting may have also contributed to the overall low agreement rates for these food items. For example, canned vegetables and processed fish or seafood tend to be stored in the pantry with other shelf stable products, where they could be inadvertently hidden or partially blocked in the photo, making it difficult to be seen by the reviewers. Furthermore, given that the Dietary Guidelines place legumes in both vegetable and protein categories [26], it is reasonable to acknowledge that study participants might conflate or confuse canned vegetables with processed legumes. As a category, other vegetables could have been overreported if participants indicated its presence in two categories (e.g., lettuce is a green leafy vegetable but is not always dark green, so some varieties might have been categorized as “other” vegetables). Participants were not asked to specify the food being referred to.

In photos of freezer foods provided by the participant, various types of meat were seen stored in bags or containers or wrapped in butcher paper which made identification of fresh fish or seafood difficult for the reviewers. The fresh or dried legumes and processed legumes could have been miscategorized by participants who did not realize that cans or cartons of beans (ready to heat) are processed or that bags of dried beans are not. Participants were instructed to report on foods stored in kitchen area only. Still, that reviewer–participant agreement was low for water/carbonated water could be explained if participants stored bottled water in places other than the kitchen area but included the water on their HFI checklist.

Adjustments to the photo instructions could help moderate some of these issues. For example, asking participants to label frozen meats or rearrange canned foods for label visibility could improve item recognition with minimal additional effort by the participant. In addition, prior to additional testing, examples of canned vegetables and processed legumes, or a description of a whole grain food could be added to the HFI to help participants select the correct food category. The item “other vegetables” is not critical for all purposes. It could be removed or included with an option for the participant to indicate which vegetable is being referred to. These suggested modifications target probable bases for the reviewer–participant discrepancies in Table 3. Implementation of these modifications could effectively improve concurrent validity and integrity of the photo method and enhance the quality of the information collected through either the photo method or the HFI checklist.

Importantly, as a means of submitting this household food information, the digital photo method was preferred to the checklist by almost 80% of the study population. Reasons for this preference were not part of this study. However, the use of food inventories is typically tedious and burdensome for the participant [27].

The photo method appears to reduce this burden and is therefore likely to increase reporting rates and data quality. This method can also accommodate the strong influence of culture and ethnicity on food choices [16]. It is likely that households with different ethnic backgrounds and social groups will have different food preferences. The digital photo method can be an easier and more inclusive approach to capturing data on the variety of foods in the home. Another advantage of this method is its application to the evaluation of nutrition education strategies that promote food behaviors associated with weight management, chronic disease prevention, and food safety. The photo method can capture data on the type of food as well as the general location of the food in the home; however, additional studies are needed to determine whether the method can also pick up changes in food placement within the refrigerator, freezer, and pantry.

This preliminary validation study has several limitations. The 53-participant study sample, though adequate in size for this type of study, did not adequately represent the regional population and cannot be generalized to the more comprehensive group of adults. The feasibility and outcome of this approach with a more diverse participant group and in more diverse communities still needs to be investigated. The photo method shifts a large part of the reporting burden from the participant to the researcher; it would be an impractical approach for large population studies or studies monitoring all foods and food products in the home. It is also possible that the preference for the photo method is attributable to its novelty or to the ease and comfort of digital technology among this young adult study population, in which case, preference, though not the validity, might change with time or target population.

## 5. Conclusions

The digital photo method was developed as a response to a need for an assessment tool that could capture the foods and the specific placement of these foods within the HFE. A practical contribution of this method is that it can also be used as an alternative to the traditional home food inventory, for assessment of other small or mid-size programs that target the home food environment. As conducted, the results of this preliminary study indicate that the photo method is valid and is the preferred method for reporting on the presence of foods in the home. Next, this photo method can be tested for sensitivity to determine change in food placement and among a more demographically diverse participant group.

This is the first time a digital photo method has been validated to replace a traditional home food inventory to assess the presence of foods and food items in the kitchen area. Minor revisions to the participant photo instructions are expected to improve clarity and reduce misinterpretation for the reviewer. Still, the photo method can be used effectively and advantageously to collect quality data.

## Figures and Tables

**Table 1:** Home food inventory (HFI) items as listed in participant survey.

Item Number	Item Category
1	Fresh fruit
2	Canned fruit
3	Dried fruit
4	Frozen fruit
5	Cut, washed, and ready-to-eat fruit
6	Canned vegetables
7	Fresh dark green vegetables
8	Fresh orange vegetables
9	Fresh yellow vegetables
10	Fresh red vegetables
11	Frozen vegetables
12	Cut, washed, and ready-to-eat vegetables
13	Other vegetables
14	Fresh meat
15	Processed meats
16	Fresh or frozen fish or seafood
17	Processed fish or seafood
18	Eggs
19	Plant proteins
20	Protein powders
21	Milk
22	Yogurt
23	Cheese
24	Rice
25	Pastas
26	Flour or corn meal
27	Whole grains
28	Ready-to-eat breakfast cereal
29	Bread
30	Fresh or dried legumes
31	Processed legumes
32	Leftovers
33	Soups
34	Nuts
35	Nut butters
36	Fats
37	Oils
38	Salty snacks
39	Sweet snacks
40	Candy/chocolate
41	Frozen desserts
42	Fruit or vegetable juice
43	Non-carbonated sweetened drinks or powders
44	Sodas
45	Water/carbonated waters
46	Alcoholic beverages

**Table 2:** Demographics of study participants.

Variables	Number of Participants (n = 53)	Total Percentage (%)
**Gender**		
Female	36	68%
Male	17	32%
**Age (years)**		
18-34	48	90.5%
35-50	4	7.5%
51-64	1	1.9%
**Ethnicity**		
Hispanic or Latino	38	71.7%
White	7	13.2%
Asian	5	9.4%
Black or AA	2	3.7%
Not sure/prefer not to say	1	2.0%
**Education**		
Currently in college	34	64.2%
4-year degree or more	17	32.1%
Associate degree, diploma, or certificate	2	3.7%
**Neighborhood**		
Suburban/Urban	51	96.2%
Rural	2	3.8%
**Food Security**		
Enough of the kinds of food they want to eat	40	75.5%
Enough but not always the kinds of food they want to eat	13	24.5%

**Table 3:** Types of discrepancies and number of occurrences for food items with kappa < 0.04.

HFI ^a^ Number	HFI Food Item ^a^	Agreement Discrepancy Type		
		Food Item Reported by Participant but Not Seen by Reviewers	Food Item Seen in Photos by One or Two Reviewers but Not Reported by Participant ^b^	Food Item Seen in Photos by All Three Reviewers but Not Reported by Participant ^b^
		Number of Occurrences		
6	canned vegetables	12	2	4
13	other vegetables	6	0	2
16	fresh fish or seafood	16	4	2
17	processed fish or seafood	12	3	1
27	whole grains	8	3	8
30	fresh or dried legumes	16	0	2
31	processed legumes	13	4	5
35	nut butters	7	3	7
39	sweet snacks	6	5	5
43	non-carbonated sweetened drinks	9	7	9
45	water/carbonated waters	10	3	2

^a^ Item numbers correspond to HFI items in Graph 2. ^b^ These two columns represent independent unduplicated measurements. Note: Shaded rows indicate the items most frequently seen by the reviewers and least frequently reported by the participant.

## Data Availability

The data could be made available through the corresponding author.
